# A Coded Structured Light System Based on Primary Color Stripe Projection and Monochrome Imaging

**DOI:** 10.3390/s131013802

**Published:** 2013-10-14

**Authors:** Sandro Barone, Alessandro Paoli, Armando Viviano Razionale

**Affiliations:** Department of Civil and Industrial Engineering, University of Pisa, Largo Lucio Lazzarino, n.1, 56126 Pisa, Italy; E-Mails: s.barone@ing.unipi.it (S.B.); a.razionale@ing.unipi.it (A.V.R.)

**Keywords:** 3D shape acquisition, texture reconstruction, Coded Structured Light, De Bruijn pattern

## Abstract

Coded Structured Light techniques represent one of the most attractive research areas within the field of optical metrology. The coding procedures are typically based on projecting either a single pattern or a temporal sequence of patterns to provide 3D surface data. In this context, multi-slit or stripe colored patterns may be used with the aim of reducing the number of projected images. However, color imaging sensors require the use of calibration procedures to address *crosstalk* effects between different channels and to reduce the chromatic aberrations. In this paper, a Coded Structured Light system has been developed by integrating a color stripe projector and a monochrome camera. A discrete coding method, which combines spatial and temporal information, is generated by sequentially projecting and acquiring a small set of fringe patterns. The method allows the concurrent measurement of geometrical and chromatic data by exploiting the benefits of using a monochrome camera. The proposed methodology has been validated by measuring nominal primitive geometries and free-form shapes. The experimental results have been compared with those obtained by using a time-multiplexing gray code strategy.

## 1. Introduction

In the last few years, an attractive research area in measurement science has been the 3D shape acquisition using vision technologies and Coded Structured Lighting (CSL) [1]. The simplest optical configuration of a CSL system includes a calibrated projector-camera pair. Light stripe patterns are deformed by object geometries and imaged by the camera. Projected and acquired patterns are then matched in order to recover surface shape information by means of triangulation procedures [2,3]. In particular, CSL systems exploit the projection of either a single pattern or a temporal sequence of patterns, which univocally determine a pixel matching code word.

Generally, CSL techniques may be classified into *continuous* and *discrete coding* methods.

*Continuous coding* techniques are characterized by smooth profiles where each pixel is addressed by code word based on gray intensity information. This peculiarity assures dense reconstructions to detriment of high sensitivity to noise and low sensitivity to surface changes, due to short distances between code words of adjacent pixels.

*Discrete coding* methods use binary fringe patterns having a digital profile with the same value for the region represented by the same code word. These techniques provide discrete coded regions, whose size determines the measurement resolution [4]. Moreover, *discrete coding* techniques can be based on either space- or time-multiplexing procedures. Space-multiplexing techniques code the scene by projecting a single pattern and analyzing the neighborhood of a given feature, whereas time-multiplexing techniques create the code word by projecting a temporal sequence of patterns.

In the field of *discrete space multiplexing*, a major group is focused on the definition of colored multi-slit or stripe patterns [5,6]. The advent of color CCD cameras and multimedia projectors has allowed the development of methods using color fringes with the aim of reducing the number of projected patterns towards one-shot techniques [7]. A color image contains red, green and blue channels, which can be used to code fringe patterns thus carrying three times information than a single gray image. Moreover, the larger vocabulary of color patterns becomes a crucial factor when absolute coding based on local neighborhood is required. However, mostly used color imaging sensors are affected by coupling effects between color channels (*crosstalk*), which require a color calibration procedure in order to minimize their influence on the measurement results [8]. Moreover, the albedo of the target objects and the ambient light may distort the stripe patterns thus requiring adaptive color calibration procedures [9]. In addition, chromatic aberrations of the lens are unavoidable due to the different refractive indices at different wavelengths. In particular, transversal chromatic aberration causes different projections of the same target point on the camera sensor plane [10]. So far, correction models for chromatic aberrations have been developed with the aim at obtaining superior quality color images, without considerations to the exact feature localization, which instead affects 3D surface reconstructions. In this context the use of a monochromatic camera rather than a color camera would lead some advantages. Monochromatic cameras are intrinsically more accurate than color cameras since the use of a single sensor rather than three sensors avoids crosstalk effects and edge artefacts. Moreover they have generally higher spatial resolution than color cameras [11], which can be exploited to increase geometric accuracy for measurement applications. However, the use of single channel cameras prevents from obtaining chromatic information associated to the reconstructed points, unless multispectral sensors, based on interference filters, are used [12].

In this paper, an edge-based reconstruction process has been developed to provide 3D surface and chromatic data by using a single channel camera and a small set of binary patterns. The methodology exploits a calibrated *color stripe projector*—*monochromatic camera* pair. The measurement process is based on a *discrete coding* method, which combines spatial and temporal information through the projection of three binary patterns. In particular, the space-multiplexing is conceptually performed by referring to a colored multi-slit pattern composed with a *De Bruijn* sequence. Practically, the color-encoded pattern is reconstructed by imaging three sequential multi-slit binary patterns with bright stripes corresponding to the three primary colors: cyan, magenta and yellow. Inverse intensity levels of the three coding patterns are also generated and projected in order to improve the stripe edge detection process.

Usually, within the scientific community two main different strategies are followed: (1) the projection of multi-colored stripes combined with a color camera; (2) the projection of monochromatic patterns combined with a single channel camera. The proposed methodology describes a hybrid solution which integrates the major accuracy obtainable by a monochromatic camera, the possibility to exploit the projection of direct and inverse intensity level patterns, and the reconstruction of consistent chromatic information even by using a single channel camera and a small set of binary patterns.

The effectiveness of the proposed methodology has been proved by measuring nominal primitive geometries and free-form shapes. The experimental results have been compared with those obtained by using a time-multiplexing gray code strategy as ground truth.

## 2. Materials and Methods

### 2.1. Hardware Set-Up

**Figure 1.**
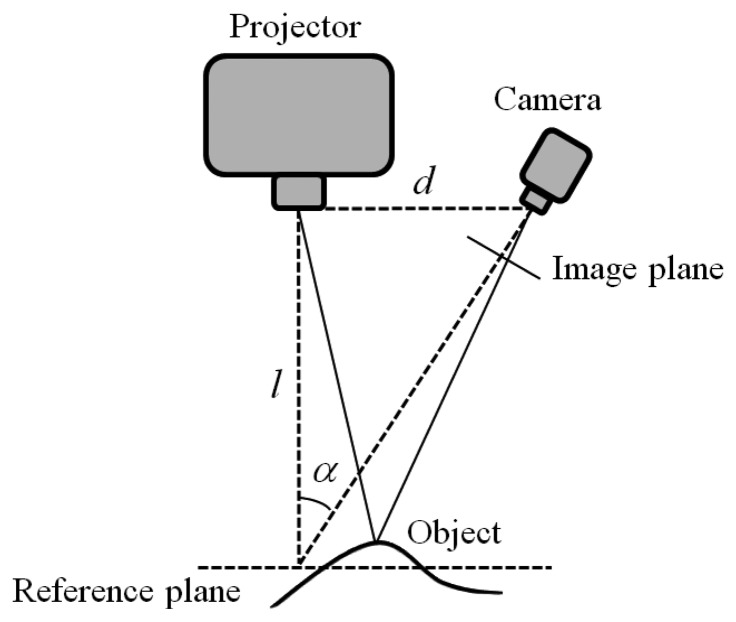
Optical set-up of the measurement system based on multi-slit pattern projection.

The 3D surface and chromatic reconstruction process has been developed using an optical system composed of a digital camera with a Sony ICX205AL CCD sensor (½ inch diagonal with a resolution of 1,280 × 960 pixels at maximum frame rate 15 fps), and a multimedia white light DLP^®^ projector based on a DMD display with a XGA resolution (1,024 × 768 pixels). The imaging devices are mounted on a rigid frame assuring an angle of about 30° between the respective optical axes and a baseline of about 300 mm for a working volume of 100 mm × 80 mm × 80 mm (width × height × depth) (Figure 1).

A calibration procedure is required to establish the values of the parameters describing the physical behavior of the optical devices (intrinsic parameters) and their relative poses (extrinsic parameters). In this work, a pinhole camera model has been used [13,14]. The model includes parameters for focal length, principal point, skew coefficient, radial and tangential distortions. The camera parameters are obtained by correlating the coordinates of known markers located on a calibrating plate with the corresponding coordinates on the camera image plane. Centre points of white circles made on a mylar sheet fixed onto a glass plate have been considered as reference markers. The projector is modeled like an inverse camera, exploiting its capability to generate both coded vertical and horizontal fringes and assuming that the camera has been previously calibrated by using the same reference points (same positions of the calibrating plate) [15].

### 2.2. Measurement Principles

**Figure 2.**
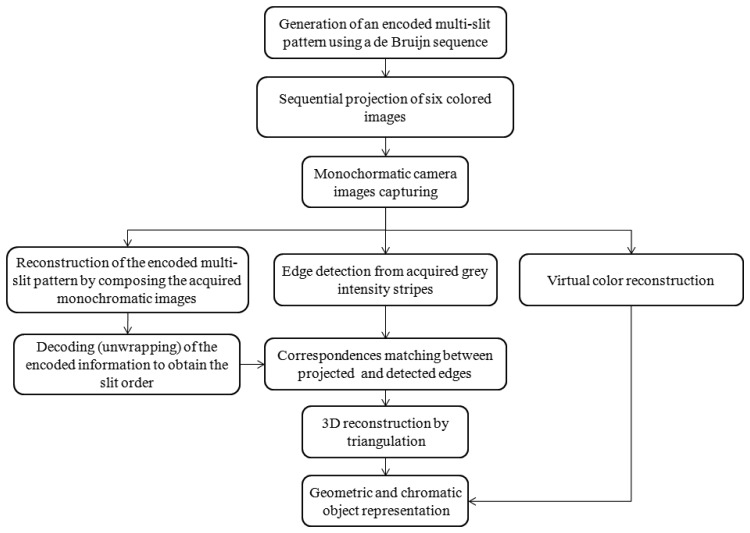
Flowchart representation of the proposed 3D measurement technique.

The proposed 3D measurement technique has been developed by integrating a virtual color encoding approach, based on a De Bruijn sequence, with an edge-based reconstruction performed on multi-slit patterns. A total of six images, containing vertical slits (stripes), are projected and acquired by the monochromatic camera. The acquired images are then used to recover the spatial encoded information, to detect the edge points for the triangulation process and to reconstruct a virtual RGB image of the scene which is used to associate chromatic information to each point. The whole procedure is schematized in the flowchart of Figure 2.

#### 2.2.1. The Pattern Projection Strategy

**Figure 3.**
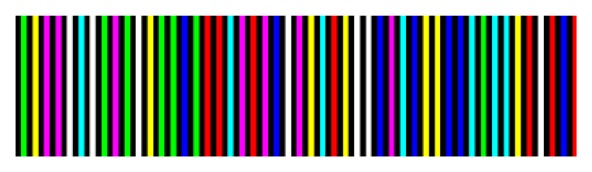
Original color-encoded De Bruijn sequence.

**Figure 4.**
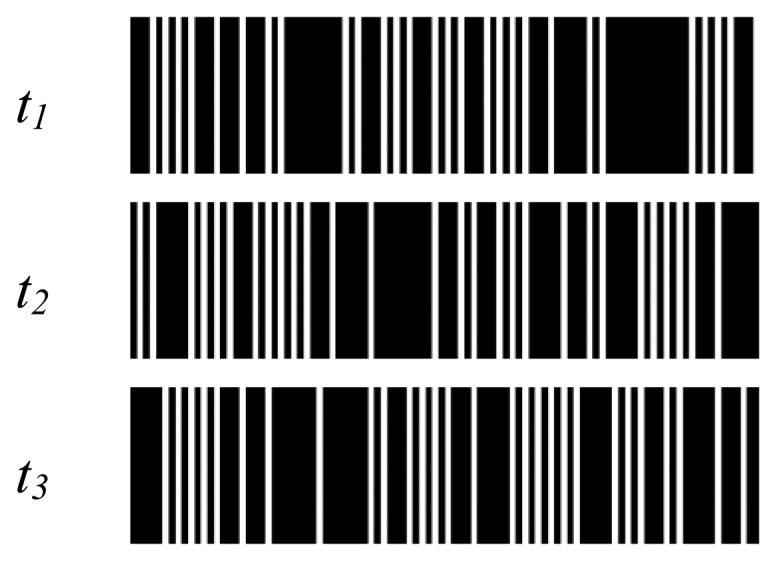
Multi-slit binary patterns used for the De Bruijn pattern reconstruction.

**Figure 5.**
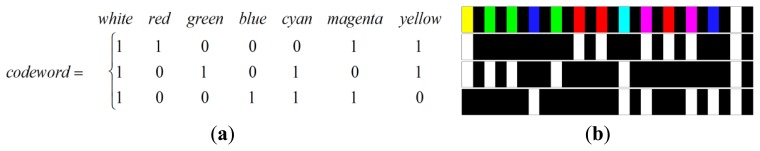
(**a**) The 7 different codeword generation, (**b**) Reconstruction detail of the original color-encoded De Bruijn sequence.

A striped color pattern is conceived on the basis of a De Bruijn sequence, which codes the position of a single stripe in the image plane by its color value and the color of the surrounding fringes. The coding strategy has been implemented by using *n* = 7 different code words which can be described by the white color and 6 other different color values (red, green, blue, cyan, magenta and yellow). Bright stripes are separated by black gaps (Figure 3), which allow two consecutive slits to have the same code word. The colored De Bruijn pattern, represented in Figure 3, is not directly projected onto the object surface, but is encoded by using the gray code principle through the sequential projection of three monochromatic striped images (Figure 4). These images may be also seen as the primary colors into which the original De Bruijn image can be decomposed. For example, each red stripe of Figure 3 is obtained by sequentially projecting a white stripe and two black stripes, while each yellow stripe is obtained by sequentially projecting two white stripes and one black stripe (Figure 5).

The De Bruijn sequence is defined in order to obtain a window property of *m* = 2 slits so that every sub-sequence of two code words appeared only once all over the scene. Three different multi-slit patterns are created and sequentially projected by the multimedia projector. This methodology codes *l_v_* = 2*n^m^* lines (for each stripe two edges can be detected since black gaps have been introduced between stripes) and *n_p_* = *r_vc_* × *l_v_* encoded points, where *r_vc_* is the vertical resolution of the camera.

The stripes edge detection is further improved by generating and projecting the negative intensity profiles of the three coding patterns. This solution increases robustness against differences between the width of bright stripes and black gaps, which may cause additional bias in stripe edge position [16]. Moreover, the projection of positive and negative intensity profiles minimize the influence of the wavelength dependence of the sensor on the edge detection.

The projection of monochrome stripes allows the measurement of geometrical information. In this paper, the reconstruction process has been adapted to contextually recover colored texture data. In particular, the scene's chromatic information is estimated by assigning a primary color (cyan, magenta or yellow) to the bright intensity regions of the positive and negative projected patterns.

Let *S* = {1, …, *n^m^*} be the set of stripe indexes. Then, the intensity profile of the each pattern image is defined as a flat function, which alternates black and color intensity vertical stripes as follows:

(1) $$P_{p}\;{\left(x\right)}_{i}=\{\begin{array}{cccccc} 0 & if\;\left(\left(x/w-1\right)\mathrm{mod}w\right) & \mathrm{mod} & 2 & < & 1\\ {\text{seq}}_{db}\;\cdot \;{\text{col}}_{i} & if\;\left(\left(x/w-1\right)\mathrm{mod}w\right) & \mathrm{mod} & 2 & \geq & 0 \end{array}\;i=1,2,3$$

where *seq_db_* is the ordered vector (having *n^m^* length) of the De Bruijn sequence, *col_i_* = cyan, magenta, yellow for *i* = 1, 2, 3, respectively, and *w* represents the pixels width of each fringe (*w* = 10 pixels).

**Figure 6.**
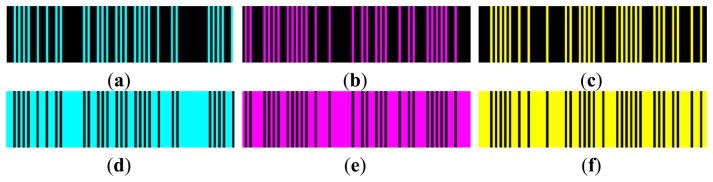
(**a**–**c**) Projected multi-slit intensity patterns relative to relation (1) along with their negative versions (**d**–**f**).

Figure 6 shows the three multi-slit intensity patterns along with their negative versions used for the sequential projection. The methodology allows 3D surface and chromatic reconstructions by only using six images.

### 2.3. Stripes Segmentation and Decoding Process

The projected vertical stripes require a decoding process through horizontal scan-lines. In particular, an accurate sub-pixel edge detection is obtained by processing image differences defined as:

(2) $$\begin{array}{c} D{\left(x,y\right)}_{1}=P_{p}{\left(x,y\right)}_{1}-P_{n}{\left(x,y\right)}_{1}\\ D{\left(x,y\right)}_{2}=P_{p}{\left(x,y\right)}_{2}-P_{n}{\left(x,y\right)}_{2}\\ D{\left(x,y\right)}_{3}=P_{p}{\left(x,y\right)}_{3}-P_{n}{\left(x,y\right)}_{3} \end{array}$$

**Figure 7.**
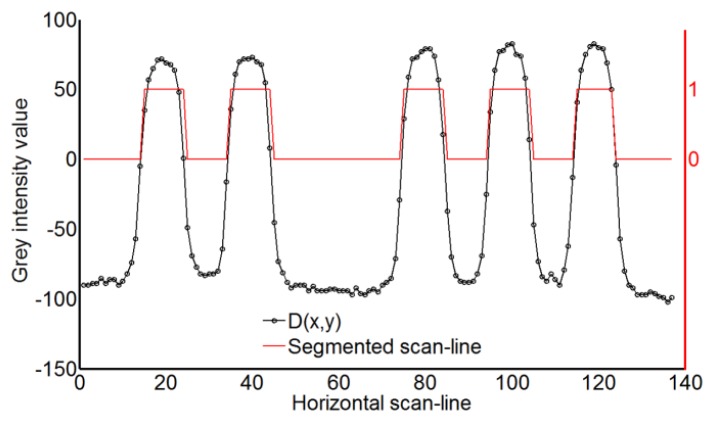
Gray intensity levels along a horizontal scan-line together with the segmented horizontal scan-line.

**Figure 8.**
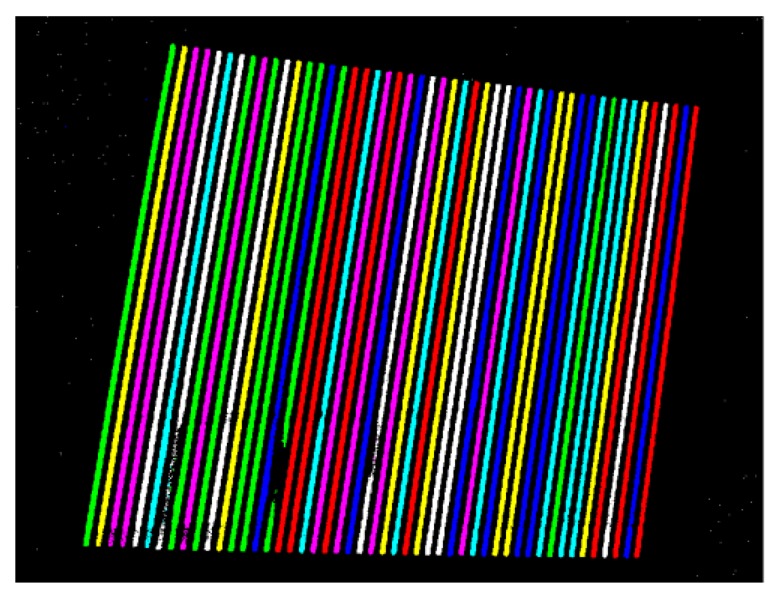
Example of reconstructed color encoded pattern for the Macbeth Color Checker acquisition.

Stripe edges are detected by computing zero-crossings of the resulting images by linear interpolation. Stripes binarization is performed for each pattern by a thresholding process with a zero threshold value (Figure 7). The code word associated to each segmented stripe (Figure 8) is then given by the temporal binary coding in accordance with Figure 5.

Correspondences between detected and projected stripes are identified by matching each subsequence of two color stripes with the ideal encoded sequence. Incorrect labeling and/or decoding due to occlusions, shadows or surface discontinuities are minimized, even in presence of abrupt surface depth variations, since the minimal window property (*m* = 2) is used.

The 3-D coordinates of the observed scene points (*point cloud*) are finally computed by triangulation using the ray-to-plane intersection, considering that the geometry of the hardware set-up, the camera ray direction and the vertical plane equation of the corresponding stripe are known.

### 2.4. Chromatic Image Reconstruction

The chromatic image of a target object is generated from three monochromatic blank images (cyan (C), magenta (M) and yellow (Y)) obtained by summing the respective positive and negative images acquired by the camera:

(3) $$\begin{array}{c} C(x,y)=P_{p}{\left(x,y\right)}_{1}+P_{n}{\left(x,y\right)}_{1}\\ M(x,y)=P_{p}{\left(x,y\right)}_{2}+P_{n}{\left(x,y\right)}_{2}\\ Y(x,y)=P_{p}{\left(x,y\right)}_{3}+P_{n}{\left(x,y\right)}_{3} \end{array}$$

where *P_n_*(*x*,*y*)*_i_* (with *i* = 1,2,3) represent the negative intensity profiles of the each pattern image. Images (3) are then used to estimate the chromatic information relative to the acquired target surfaces. The procedure can be schematized as follows:

- Detection and removal of saturated pixels (if existing);
- Normalization to the interval [0,1] in order to stretch gray intensity levels into the whole intensity range for each of the three channels;
- Conversion to RGB color space through the computation of red (R), green (G) and blue (B) components from CMY values by:
  (4) $$\begin{array}{c} R(x,y)=[(M(x,y)+Y(x,y))-C(x,y)]/2\\ G(x,y)=[(C(x,y)+Y(x,y))-M(x,y)]/2\\ B(x,y)=[(C(x,y)+M(x,y))-Y(x,y)]/2 \end{array}$$
- Creation of a virtual color image by composing the RGB channels.

The chromatic information is then automatically mapped on the 3D model by assigning an RGB triplet to each measured point.

**Figure 9.**
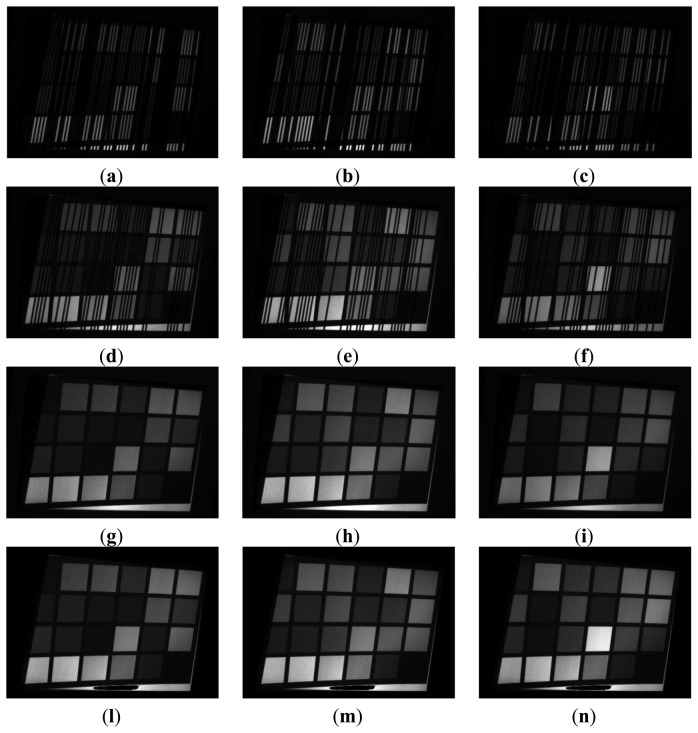
Virtual color reconstruction of a Macbeth Color Checker. (**a**–**c**) Monochromatic camera acquisition of positive projected patterns, (**d**–**f**) Monochromatic camera acquisition of negative projected patterns, (**g**–**i**) CMY channels obtained by applying Equation (3), (**l**–**n**) Effects of the removal of saturated pixels and stretching process.

**Figure 10.**
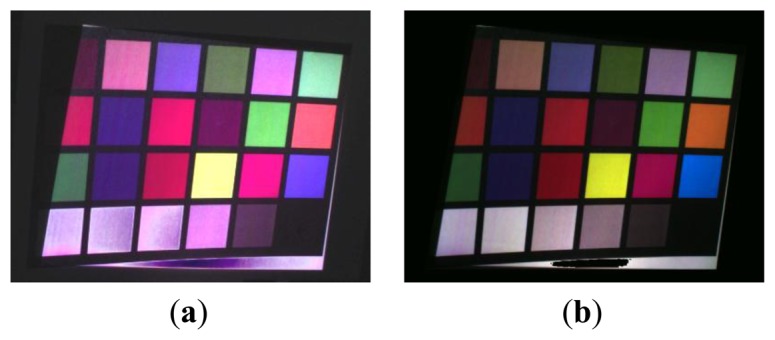
Chromatic reconstruction obtained by composing RGB values obtained by Equation (4) before (**a**) and after (**b**) the removal of saturated pixels and the normalization of gray intensity values.

The whole process is described in Figures 9 and 10, which present the color reconstruction of a Macbeth Color Checker [17]. Figure 9a–c and Figure 9d–f show the sequential monochromatic camera acquisitions of patterns relative to Figure 6a–c and Figure 6d–f, respectively. Figure 9g–i show the CMY channels as obtained by applying Equation (3), whereas Figure 9l–n present the same channels after removing the saturated pixels and normalizing the gray intensity levels.

Figure 10 show the results of the composition of the RGB channels obtained by applying Equation (4) before (Figure 10a) and after (Figure 10b) removing saturated pixels and normalizing the gray intensity values.

## 3. A Ground Truth Gray-Code Coding Method

**Figure 11.**
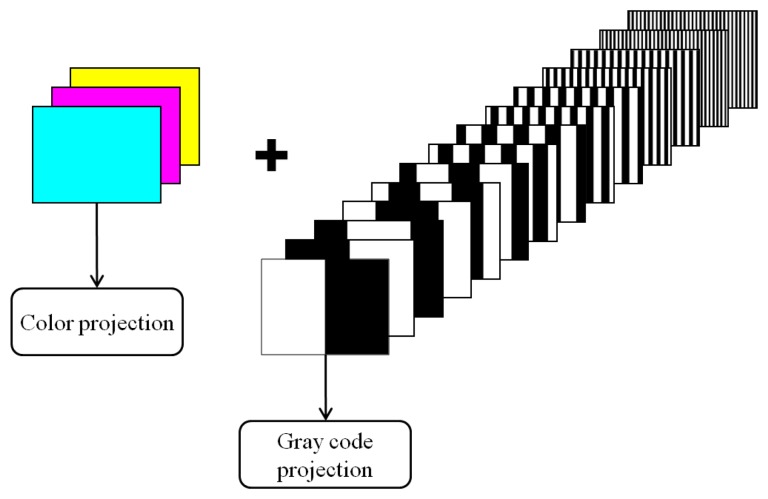
Projection scheme for the adopted gray code method.

A time-multiplexing coding strategy has been also implemented in order to create a ground truth for comparison analyses [18]. A sequence of patterns having black and white stripes, whose period is progressively halved, is projected onto the scene following a coarse-to-fine strategy. The length of the codeword is then given by *2^p^* bits, where *p* is the total number of projected patterns. In this paper, seven patterns have been used in order to uniquely code *2^7^* different vertical lines, thus providing a comparable number of coded lines with respect to the developed methodology. For each pattern, its inverse intensity distribution is also projected. Moreover, the gray code sequence is preceded by the projection of three blank images providing the primary colors cyan, magenta and yellow, respectively, in order to allow the reconstruction of the chromatic information (Figure 11).

This methodology is still considered the most accurate CSL measurement strategy since each detected vertical line is only encoded through a temporal sequence pattern without requiring any spatial information. However, robustness and accuracy of this technique are counterbalanced by the high number of patterns which must be projected. The strictly temporal binary coding strategy used as ground truth for the experimental validation is based on the projection of 17 different patterns.

## 4. Experimental Results

The proposed CSL system has been validated by acquiring two primitive shapes and a complex anatomical model. In particular, a plane and a cylinder (nominal diameter *d* = 80 mm) have been firstly measured in order to test metric accuracy and robustness to surface reflectance properties in comparison with the ground truth coding methodology. An anatomical model has been finally reconstructed with the aim at evaluating the feasibility of the proposed technique in measuring both geometric and chromatic information of non collaborative shapes.

### 4.1. Nominal Shape Measurement

**Figure 12.**
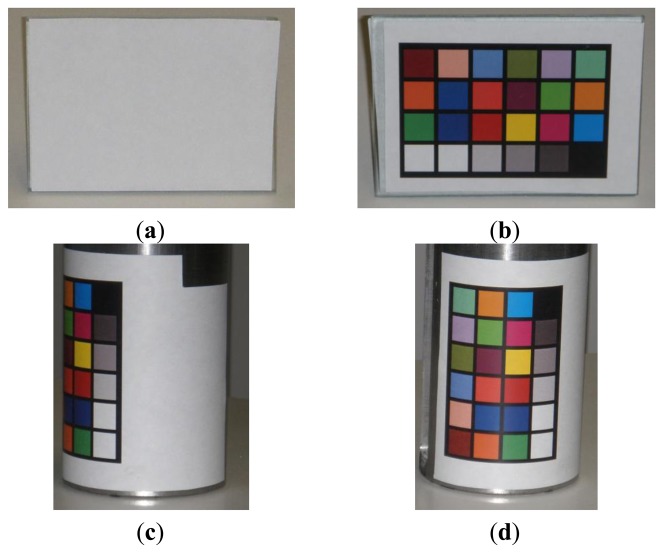
Primitive shapes used for the experimental tests: white (**a**) and Macbeth (**b**) plane, white (**c**) and Macbeth (**d**) cylinder.

Figure 12 show the primitive shapes used for the experimental tests. Planar and cylindrical surfaces have been measured with a white (Figure 12a,c) and varying colored (Figure 12b,d) background. The effect of different colors has been reproduced with a Macbeth Color Checker printed on a 0.05 mm thick paper sheet and attached onto the primitive surfaces. The reconstruction of such real target objects allows the analysis of accuracy, repeatability and sensitivity to chromatic features.

A variant of the proposed methodology has been also developed by replacing the projected primary colors, cyan, magenta and yellow with red, green and blue, respectively. Clearly, relations (4) are not applied anymore since RGB components are directly acquired by the camera. This variant, hereinafter named *RGB method* [19], has been conceived with the aim at verifying possible differences in the color reconstruction using different primary color sets.

**Figure 13.**
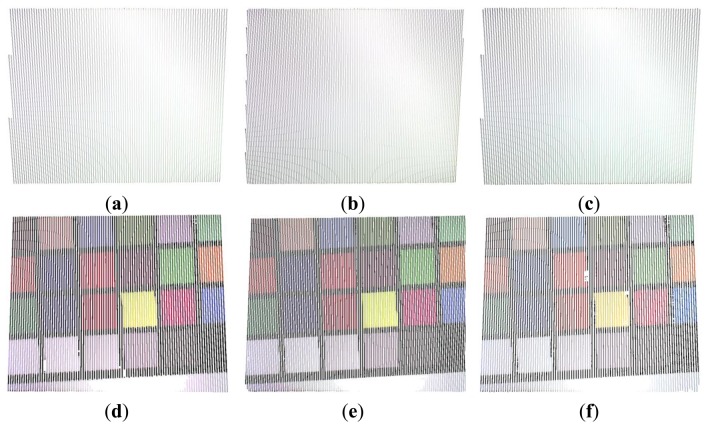
Point clouds obtained by measuring the white and Macbeth plane targets through the developed methodology (**a**,**d**), the gray code method (**b**,**e**) and the RGB variant (**c**,**f**).

**Figure 14.**
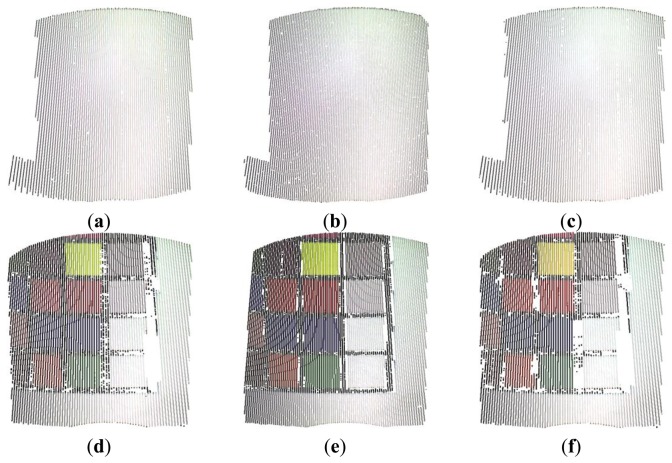
Point clouds obtained by measuring the white and Macbeth cylinder targets through the developed methodology (**a**,**d**), the gray code method (**b**,**e**) and the RGB variant (**c**,**f**).

Figure 13 show the results obtained by acquiring the white and Macbeth plane by using the developed methodology (Figure 13a,d), the gray-code method (Figure 13b,e) and the RGB variant (Figure 13c,f). Figure 14 shows the same results for the cylinder acquisition. The planar and the cylindrical surfaces have been reconstructed by point clouds including about 70,000 points and 60,000, respectively.

**Figure 15.**
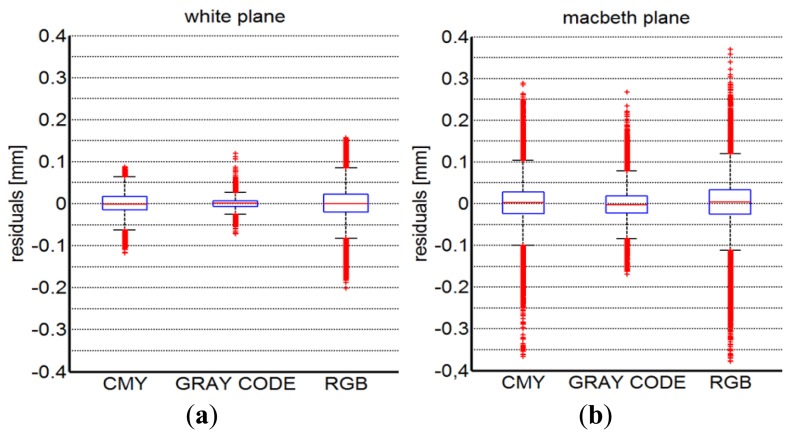
Boxplot representation of the residuals arising by best fitting the data acquired from white (**a**) and multi-colored (**b**) planar surfaces.

The measurement accuracy has been then evaluated by best fitting the acquired data with a plane and a cylinder, respectively. Figure 15a,b show a boxplot description of the residual distributions occurring by best fitting the point clouds obtained from the acquisition of the white and the Macbeth planar targets, respectively. The standard deviation of the residual best fitting errors is computed for each measurement. Moreover, five different measurements for each methodology have been carried out by slightly changing the illumination conditions (*i.e.*, variations of the camera exposure time) in order to evaluate the standard deviation variability. Mean (*μ_p_*) and standard deviation (*σ_p_*) relative to these different tests are reported in Table 1.

**Figure 16.**
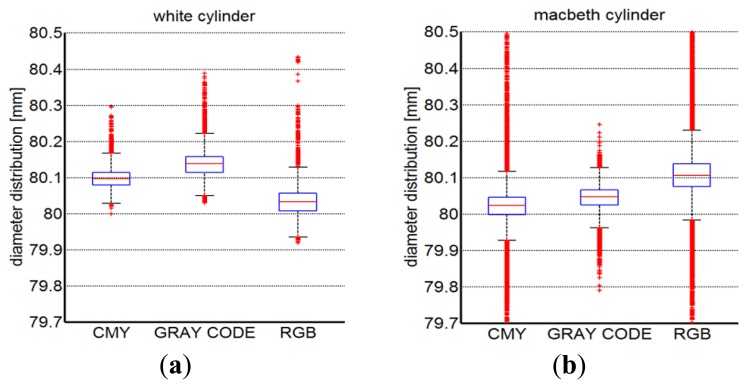
Boxplot description of the diameter results as obtained by best fitting the acquired point clouds from white (**a**) and multi-colored (**b**) cylindrical surfaces.

Similarly, the three different implemented scanning methodologies have been used to acquire the cylindrical shapes. Figure 16a,b present the boxplot descriptions of the diameter results measured by best fitting the point clouds obtained from the acquisition of white and the Macbeth cylindrical targets, respectively. The measurements variability has been evidenced by reporting in Table 2 the mean diameter values (*μ_d_*) along with their relative standard deviations (*σ_d_*) over five different measurements.

The results obtained by adopting the developed methodology are similar to those obtained by using the ground truth methodology both in terms of spread of the residuals (height of the boxes in the boxplot representations) and repeatability. The differences between mean values of the residual distributions of the best fitting plane process differ for about 10^−2^ mm between the developed and the ground truth methodology. Similar differences can be observed also for the cylindrical shape acquisition. Slightly higher differences occur for colorful nominal shapes (in particular for the cylindrical shape).

The best accuracies, with a minor number of outliers (red crosses), are obtained for the time multiplexing gray code methodology. This is much more evident when colored objects are measured since the projection of black and white fringes produces higher contrast ratios for the edge detection task than primary colors projection. The projection of RGB color stripes causes greater disturbs than those occurring with CMY primary colors. Actually, this is an expected result since the RGB projection only uses a third part of the white light intensity thus producing poorer reconstructions especially when dark surfaces are acquired.

The accuracy obtained in the color reproduction, with respect to the ground truth methodology, has been evaluated by analyzing the Macbeth plane acquisition. The virtual color image, reconstructed by composing RGB channels (Equation (4)), has been converted into HSV color space. This color space separates the color information (Hue, H) from the information of color purity (Saturation, S) and intensity (V). Hue is defined as an angle in the range [0, 2π] relative to the red axis with red at angle 0, pure green at 2π/3, pure blue at 4π/3 and red again at 2π [20].

**Figure 17.**
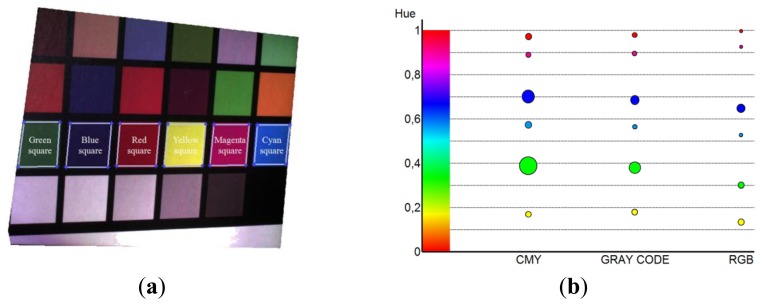
(**a**) Macbeth Color Checker patches used to test the color reconstruction effectiveness and (**b**) the bubble chart of the mean hue values (circle centers) and standard deviation values (circle radii) relative to 6 different colored patches.

Hue values of the Macbeth Color Checker patches (Figure 17a) have been normalized in the range [0,1] and used to analyze the effectiveness of the color reconstruction process. Figure 17b reports the bubble chart of the mean hue values (circle centers) and the standard deviation values (circle radii) relative to six different colored patches acquired by using the three implemented methodologies. The repeatability of the mean hue values obtained for the six patches has been estimated by carrying out five different measurements. Mean and standard deviation values of these tests have been summarized in Table 3.

The comparison between the different techniques highlights a greater spread of the hue values for the CMY method if compared with both gray code and RGB techniques. Gray code method assures a cleaner color reproduction since the projection of the three primary colors is not impaired by the presence of binary fringes. In general, the smallest data scattering emerges from the RGB method since the red, green and blue components are directly acquired by the camera and not obtained by combining all the three channels (Equation (4)). Nevertheless the mean hue values for the considered patches are consistent among the proposed and the ground-truth methodology. Slight differences in the mean values can be observed with the RGB variant, even if the final chromatic perception is not affected.

### 4.2. Anatomical Surface Reconstruction

**Figure 18.**
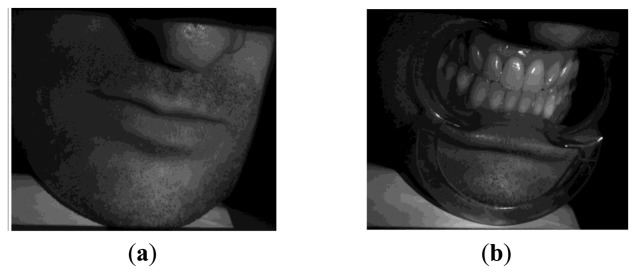
Anatomical surfaces regarding a human mouth (**a**) and the relative dental arches (**b**).

**Figure 19.**
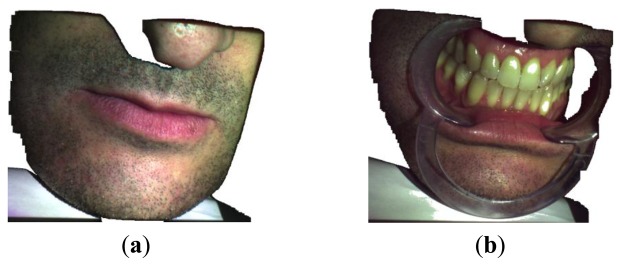
Chromatic images as obtained by the proposed methodology for the human mouth (**a**) and the relative dental arches (**b**).

The developed methodology has been further experienced in the acquisition of free-form surfaces with special regard to the reconstruction of anatomical shapes (Figure 18). Figure 19 shows the virtual chromatic images obtained by composing the red, green and blue components as obtained from Equation (4) in the acquisition of a human mouth (Figure 19a) and the relative dental arches in occlusion condition (Figure 19b).

**Figure 20.**
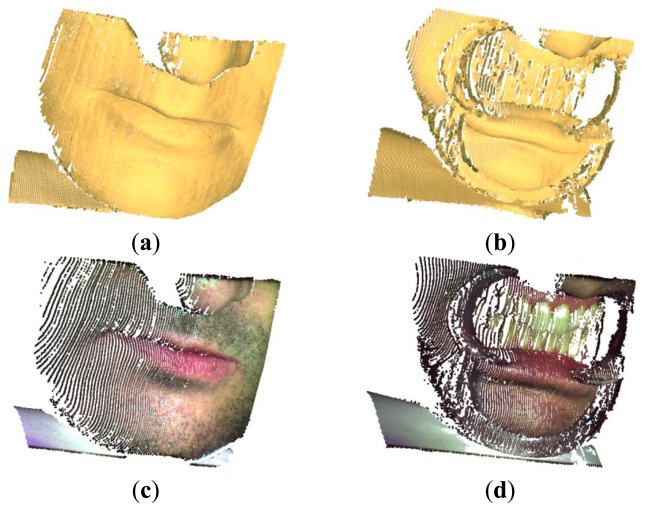
Reconstructed 3D point clouds of a human mouth and the relative dental arches without (**a**,**b**) and with the chromatic information associated to the anatomical shape (**c**,**d**).

Figure 20a,b show the final 3D representations (*point clouds*) obtained by the proposed CSL methodology for the mouth (about 67,000 points) and the dental arches (about 55,000 points), respectively. The chromatic data have been finally mapped to the respective anatomical geometries (Figure 20c,d). Holes occurring near the nose (Figure 18a) and mouth areas (Figure 18b) are due to optical occlusions and limited imaging field of the sensors. More views can be collected by moving the acquisition system and/or the anatomical surface and integrated into a common reference frame in order to reconstruct the entire shape. The results demonstrate that the color reproduction greatly enhances the perception of anatomical shapes with respect to models only containing geometrical information.

## 4. Discussion and Conclusions

In this paper, a novel Coded Structured Light technique has been developed with the aim at obtaining 3D geometrical reconstructions, also including chromatic attributes, by using a small set of stripe patterns. The proposed coding strategy is focused on the minimization of the number of projected patterns, with respect to standard time multiplexing gray code techniques, by preserving at the same time both resolution and accuracy. The methodology demonstrated to be geometrically robust even for colorful objects.

The projection of positive and negative patterns automatically overcomes the problem of the proper selection of the threshold value to be used in the edge detection task, which typically affects reconstruction accuracies when only positive images are used. Stripe edges could be determined by computing zero-crossing of the second derivative of the original images in the orthogonal direction with respect to the stripes. However, the filter size should be defined in order to be comparable to the edge width, which may vary within the image space.

The proposed coding strategy makes the methodology robust against illumination condition as well as surface reflectance properties. The projection of patterns with bright stripes lighted up by cyan, magenta and yellow, rather than red, green and blue, assures good accuracies, which get closer to those obtained by using a time multiplexing gray code strategy, making also feasible the reconstruction of non collaborative surfaces (*i.e.*, dark surfaces, human teeth). Moreover, the use of primary colors as cyan, magenta and yellow allows the reproduction of chromatic attributes of colorful 3D objects even by using a single channel monochromatic camera. The color reconstruction could be further improved in terms of chromatic reliability by a white-balancing calibration procedure. However, a coarse reproduction of chromatic attributes could be still useful for many issues. In particular, biomedical applications require 3D geometrical and chromatic data for diagnostic assessments. In these cases, the proposed methodology offer an optimal trade-off between scanning process speed and measurement data attributes (robustness, accuracy, completeness). These peculiarities are very useful for reconstructing real anatomies, which generally are non static targets characterized by multicolored textures.

## Figures and Tables

**Table 1.** 3D geometric accuracies for planar surfaces: mean values (*μ_p_*) and standard deviation (*σ_p_*) of the residuals standard deviations obtained for five different measurements.

	**White Plane**		**Macbeth Plane**	
	*μ_p_* (mm)	*σ_p_* (mm)	*μ_p_* (mm)	*σ_p_* (mm)
**CMY**	0.022	0.0007	0.047	0.002
**GRAY CODE**	0.012	0.00008	0.034	0.0005
**RGB**	0.043	0.0002	0.073	0.006

**Table 2.** 3D geometric accuracies for cylindrical surfaces: mean diameter values (*μ_d_*) along with their relative standard deviation values (*σ_d_*) obtained for 5 different measurements.

	**White Cylinder**		**Macbeth Cylinder**	
	*μ_d_* (mm)	*σ_d_* (mm)	*μ_d_* (mm)	*σ_d_* (mm)
**CMY**	80.167	0.029	80.004	0.055
**GRAY CODE**	80.137	0.0317	80.017	0.0387
**RGB**	80.052	0.0592	80.057	0.077

**Table 3.** Assessment of the chromatic reconstruction process: mean hue (*μ*) and standard deviation (*σ*) values of the six colored patches as obtained by considering 5 different measurements.

	**Red Square**		**Green** **Square**		**Blue Square**		**Yellow** **Square**		**Magenta** **Square**		**Cyan** **Square**	

	***μ****_r_*	***σ****_r_*	***μ****_g_*	***σ****_g_*	***μ****_b_*	***σ****_b_*	***μ****_y_*	***σ****_y_*	***μ****_m_*	***σ****_m_*	***μ****_c_*	***σ****_c_*
***CMY***	0.977	0.007	0.382	0.008	0.682	0.006	0.166	0.005	0.888	0.005	0.572	0.008
***GRAY*** ***CODE***	0.982	0.003	0.366	0.011	0.668	0.010	0.181	0.009	0.894	0.004	0.564	0.006
***RGB***	0.996	0.001	0.305	0.002	0.641	0.003	0.134	0.002	0.924	0.004	0.527	0.005
***Theoretical*** ***hue value***	1		0.333		0.666		0.166		0.833		0.5	
